# Emphysema elevates the DETECT scores: impact on pulmonary hypertension screening and diagnosis in systemic sclerosis

**DOI:** 10.1093/rheumatology/keaf410

**Published:** 2025-08-01

**Authors:** Gesa M Sauer, Florian Käs, Carmen-Marina Mihai, Muriel Elhai, Rucsandra Dobrota, Mike O Becker, Sinziana Muraru, Anna-Maria Hoffmann-Vold, Oliver Distler, Cosimo Bruni

**Affiliations:** Department of Rheumatology, University Hospital Zurich, University of Zurich, Zurich, Switzerland; Department of Rheumatology, University Hospital Zurich, University of Zurich, Zurich, Switzerland; Department of Rheumatology, University Hospital Zurich, University of Zurich, Zurich, Switzerland; Department of Rheumatology, University Hospital Zurich, University of Zurich, Zurich, Switzerland; Department of Rheumatology, University Hospital Zurich, University of Zurich, Zurich, Switzerland; Department of Rheumatology, University Hospital Zurich, University of Zurich, Zurich, Switzerland; Department of Rheumatology, University Hospital Zurich, University of Zurich, Zurich, Switzerland; Department of Rheumatology, University Hospital Zurich, University of Zurich, Zurich, Switzerland; Department of Rheumatology, Oslo University Hospital, University of Zurich, Oslo, Norway; Department of Rheumatology, University Hospital Zurich, University of Zurich, Zurich, Switzerland; Department of Rheumatology, University Hospital Zurich, University of Zurich, Zurich, Switzerland

**Keywords:** systemic sclerosis, CPFE, emphysema, interstitial lung disease, DETECT score, pulmonary hypertension

## Abstract

**Objectives:**

Combined pulmonary fibrosis and emphysema (CPFE) predicts unfavourable outcomes in systemic sclerosis (SSc). CPFE and emphysema are associated with pulmonary function tests (PFTs) abnormalities. As screening algorithms for pulmonary hypertension include PFTs, we aimed to assess whether CPFE and emphysema affect the performance of the DETECT algorithm to select patients for right-heart catheterization (RHC).

**Methods:**

SSc patients from our referral centre, with available chest tomography images to identify emphysema or interstitial lung disease (ILD) and data to calculate the DETECT score were included. Baseline visit was set as the first visit with available information. Patients with any form of pulmonary hypertension at baseline were excluded. We tested the association of CPFE (and ILD and emphysema separately) with the DETECT score, DETECT score positivity and false positivity, using regression models adjusted for selected covariates.

**Results:**

Among 550 eligible cases, ILD was detected in 232 (42%) and emphysema in 59 (11%) patients, resulting in a 7% prevalence of CPFE. The DETECT score was higher and on average above the threshold for referral to RHC in emphysema and CPFE patients. After adjustment, both emphysema and CPFE predicted positive DETECT scores, while ILD did not. Nevertheless, we found no association between CPFE nor emphysema with false-positive DETECT scores.

**Conclusion:**

In SSc, CPFE and emphysema are associated with a higher and more frequently positive DETECT score. As neither CPFE nor emphysema were associated with false-positive DETECT results, our data also support the validity of the DETECT algorithm in these SSc subgroups.

Rheumatology key messages

- DETECT score is significantly elevated in combined pulmonary fibrosis and emphysema (CPFE)/emphysema patients.
- In contrast to emphysema, ILD does not contribute independently to increasing the DETECT score step 1 or step 2 values, nor the rate of DETECT positivity.
- Regardless, DETECT score is a usable tool in CPFE/emphysema SSc patients.

## Introduction

Systemic sclerosis (SSc) is a multisystem autoimmune disease characterized by fibrotic changes of different organs and tissues, leading to high morbidity and mortality. The two leading causes of death in SSc are interstitial lung disease (ILD) and pulmonary hypertension (PH) [1].

There is increasing evidence for airway disease as a further risk factor in SSc, in particular in co-existence with ILD [2]. First described in 2005, ‘combined pulmonary fibrosis and emphysema’ (CPFE) is defined as the combination of ILD in the lower and emphysema in upper lung regions [3]. The prevalence of CPFE among SSc patients has been reported to range between 7.5% and 12.5% among SSc-ILD patients [4–6]. CPFE has been associated with an unfavourable outcome in SSc, leading to reduced survival and increased emergency hospitalizations for cardio-respiratory failure, in comparison to SSc patients with isolated ILD [4, 5]. This is even more relevant within SSc PH cohorts, where CPFE represents 27% of group III PH patients, underlining the complex interaction between different parenchymal pulmonary and vascular involvements [7].

Patients with CPFE show marked changes in pulmonary function tests (PFTs), such as the diffusion capacity of the lung for carbon oxide (DLCO) and an increased forced vital capacity (FVC)/DLCO ratio [5, 6, 8]. Both parameters are closely associated with pulmonary hypertension [9, 10] and, in particular, have proven useful for the early identification of patients with pulmonary involvement and, as a result, have been incorporated into various algorithms [11–15]. Regarding PH screening, the FVC/DLCO ratio is an important component of the DETECT score, with higher values leading to a higher probability of referral to diagnostic right-heart catheterization (RHC).

Because CPFE and PH share similarities in terms of impact on survival and PFTs impairment, the usability of the DETECT score in CPFE patients [6] and, indirectly, in SSc patients with emphysema or ILD, might be questioned.

The aim of the present study was, first, to characterize CPFE/emphysema patients in our SSc cohort; second, to study the DETCT score and positivity of the DETECT score in CPFE/emphysema patients. Finally, we determined if the DETECT score was more often false-positive in the CPFE/emphysema cases, contributing to unnecessary RHC.

## Methods

For this observational cohort study, we selected SSc patients (classified according to the 2013 ACR/EULAR criteria) registered in the local database of the Department of Rheumatology, University Hospital Zurich. Patients were eligible if they presented with available chest high-resolution computed tomography (HRCT) images, as well as availability of the variables needed to calculate the DETECT score. One missing DETECT score parameter was allowed, as per algorithm validation [11]. Any form of PH diagnosed before the baseline visit represented an exclusion criterion. Given the multiorgan involvement and the complex separation of primary pathogenetic PH determinants in this population, we did not exclude patients with left heart disease (both systolic and diastolic) or other pulmonary conditions. Similarly, in line with the recent international recommendations [16] and the observational evidence from unselected patients [17], we did not exclude patients not fulfilling the original DETECT study criteria (>3 years from SSc onset, FVC >40% and DLCO <60%).

For each eligible patient, the first visit with available required information was selected as baseline visit. The diagnosis of emphysema is per definition based on HRCT, where it is characterized by irreversible enlargement of airspaces with destruction of alveolar walls [18]. For our study, we defined emphysema according to the evaluation of the assessing radiologist based on HRCT, without specification of the subtype (centrilobular, panlobular, paraseptal). Presence of ILD was defined according to the Fleischner criteria [18] by the assessing radiologist, based on HRCT images. Patients were considered as CPFE when ILD and emphysema were both present.

First, patients were grouped and compared based on the occurrence of emphysema (emphysema: yes/no) and on the combination of emphysema and ILD in four groups: negative HRCT, ILD only, emphysema only and CPFE. Finally, patients were differentiated based on DETECT score (positive if >35 points, otherwise negative) and true- *vs* false-positivity. The latter analysis compared all DETECT positive patients who underwent RHC. Patients were diagnosed with PH based on the 2022 guidelines of the European Society of Cardiology/European Respiratory Society if the mean pulmonary arterial pressure (mPAP) on RHC was >20 mmHg [16]. We considered true positive all patients fulfilling this criterion, regardless of the pathogenetic mechanisms, as the prognosis of SSc patients is negatively impacted by the presence of any PH class and its detection would lead to beneficial therapeutic interventions. Additionally, previous literature showed comparable performance of the DETECT algorithm also in patients with PH attributed to left heart or lung disease [17].

Categorical variables were presented as absolute and relative frequencies. For continuous variables, mean and standard deviation (SD) or median and interquartile range (IQR) were presented, according to data distribution. Comparison between groups was performed using the χ^2^ or the Fischer’s exact test for categorical variables, while the Student’s *t* test or the ANOVA test were used for continuous parameters.

In univariable and multivariable analysis, linear regression models were built to test the association of CPFE/emphysema with the DETECT step 1 and step 2 scores. When DETECT score step 2 positivity (defined as DETECT score >35 points) and DETECT score false positivity (DETECT score >35 points, but normal RHC haemodynamic profile) represented the dependent variable, logistic regression models were applied to quantify the association of CPFE/emphysema with it. Due to the limited number of RHC performed in our study population, only univariable analysis was applied to predict false-positive DETECT scores, while all other models included covariates selected according to literature data and expert experience. Those included age, sex, disease duration from the first non-Raynaud’s sign or symptom, digital ulcers [19], smoking exposure, left ventricular ejection fraction and diastolic dysfunction on echocardiography. The latter was defined by the reporting cardiologist during echocardiography according to recent international consensus documents [20].

For all regression models, an alternative model was tested differentiating ILD and emphysema, both being determinants of the CPFE variable. This was performed by replacing the variable ‘CPFE’ with the variables ‘ILD’ and ‘emphysema’.

Statistical analysis was performed using IBM SPSS Statistics v. 26, selecting a *P*-value <0.05 for statistical significance. Each patient included in the local database had signed informed consent for data collection and analysis for the study, approved by the Cantonal Ethic Committee Zurich (BASEC-Nr.2016–01515 and BASEC-Nr.2018–02165).

## Results

### Study population

At database censoring, 3019 visits over 20 years (2003–2023) from 796 SSc patients were available. After excluding visits with missing data, we identified 550 SSc patients with at least one eligible visit for our analysis.

The study population consisted mostly of female patients (83%), with mean age and disease duration of 56 and 9 years, respectively. Current or past smoking history was reported by 147 patients (28%). Concerning SSc features, 104 patients (19%) had the diffuse cutaneous subset, 232 (42%) had ILD on HRCT and 159 (30%) had current or previous digital ulcers. Additional clinical characteristics are presented in Table 1.

**Table 1.** Description of the study population and comparison between emphysema/ILD/CPFE groups

	All patients (*n* = 550)	Emphysema yes (*n* = 59)	Emphysema no (*n* = 491)	*P*-value^*^	HRCT negative (*n* = 295)	ILD only (*n* = 196)	Emphysema only (*n* = 23)	CPFE (*n* = 36)	*P*-value^**^
Age, years, mean ± SD	56 ± 14	62 ± 12	55 ± 15	**0.001**	54 ± 15	58 ± 14	57 ± 10	65 ± 13	**<0.001**
Sex female, n (%)	457 (83)	45 (76)	412 (84)	0.143	257 (87)	155 (79)	17 (74)	28 (78)	**0.050**
Disease duration, years, mean ± SD	9.2 ± 9.7	10.2 ± 10.2	9.1 ± 9.6	0.388	8.4 ± 8.2	10.2 ± 11.3	11.1 ± 11.1	9.8 ± 9.8	0.167
Smoking ever, *n* (%)	147/527 (28)	33 (61)	114 (24)	**<0.001**	65 (23)	49 (26)	15 (68)	18 (50)	**<0.001**
Diffuse cutaneous SSc, *n* (%)	104 (19)	12 (21)	92 (19)	0.860	30 (10)	62 (32)	1(4)	11 (31)	**<0.001**
mRSS, median (IQR)	0 (0–5)	2 (0–6)	0 (0–4)	0.083	0 (0-2)	2.5 (0–8)	1 (0–4)	3.5 (0–7)	**<0.001**
Digital ulcers ever, *n* (%)	159 (29)	25 (42)	134 (27)	**0.022**	50 (17)	84 (43)	7 (30)	18 (50)	**<0.001**
Puffy fingers, *n* (%)	381/527 (69)	37 (65)	344 (73)	0.210	189 (68)	155 (82)	14 (63)	23 (66)	**0.005**
Telangiectasia, *n* (%)	284 (52)	38 (64)	246 (50)	**0.039**	135 (46)	111 (57)	13 (57)	25 (69)	**0.012**
Arthritis, *n* (%)	98/538(18)	13 (22)	85 (18)	0.471	48 (17)	37 (19)	5 (22)	8 (23)	0.634
Esophageal symptoms, *n* (%)	279 (51)	36 (61%)	248 (50)	0.1	142 (48)	101 (52)	11 (48)	25 (69)	0.113
Renal crisis, *n* (%)	6/549 (1)	3 (5)	3 (1)	**0.019**	0	3 (2)	0	3 (8)	**0.002**
ACA, *n* (%)	245/517 (47)	24 (41)	221 (48)	0.404	182 (66)	39 (21)	14 (64)	10 (29)	**<0.001**
ATA, *n* (%)	137/543 (25)	14 (24)	123 (25)	0.874	36 (12)	87 (45)	2 (9)	12 (33)	**<0.001**
ARA, *n* (%)	68/533 (13)	9 (16)	59 (12)	0.531	32 (11)	27 (14)	5 (23)	4 (11)	0.337
Diastolic dysfunction, *n* (%)	99/466 (16)	12 (20)	87 (18)	0.584	42 (14)	45 (24)	6 (27)	6 (18)	0.054
Uric acid (mg/dl), mean ± SD	4.8 ± 1.5	5.7 ± 2.2	4.7 ± 1.3	**<0.001**	4.6 ± 1.2	4.9 ± 1.5	5.5 ± 2.4	5.8 ± 2.1	**<0.001**
FVC, % predicted, mean ± SD	93 ± 19	86 ± 19	94 ± 18	**0.001**	99 ± 16	88 ± 20	93 ± 15	82 ± 21	**<0.001**
DLCO, % predicted, mean ± SD	74 ± 22	55 ± 21	76 ± 20	**< 0.001**	82 ± 19	69 ± 21	63 ± 19	49 ± 20	**<0.001 **
FVC/DLCO, ± SD	1.37 ± 0.51	1.9 ± 1.0	1.3 ± 0.4	**<0.001**	1.26 ± 0.32	1.37 ± 0.43	1.61 ± 0.62	2.15 ± 1.07	**<0.001 **
ILD, *n* (%)	232 (42)	36 (61)	196 (40)	**0.002**	0	196 (100)	0	36 (100)	**N.A.**
NYHA, *n* (%)									**<0.001 **
I	319 (59)	22 (38)	297 (61)	**<0.001**	198 (68)	99 (51)	13 (57)	9 (26)	
II	184 (34)	21 (36)	163 (34)		84 (29)	79 (40)	9 (39)	12 (34)	
III	34 (6)	11 (19)	23 (5)		8 (3)	15 (8)	1(4)	10 (29)	
IV	7 (1)	4 (7)	3 (1)		0	3 (2)	0	4 (11)	
NT-proBNP, median (IQR)	101 (56-214)	211 (77-500)	93 (54-187)	**<0.001**	91 (51-158)	112.5 (58-268)	127 (58-362)	239 (93-645)	**<0.001**
LVEF %, mean ± SD	61 ± 5	60 ± 5	61 ± 5	0.402	61 ± 5	61 ± 5	59 ± 6	61 ± 4	0.419
Right axis deviation, *n* (%)	5 (1)	1 (2)	4 (1)	0.315	2 (1)	2 (1)	0	1 (3)	**<0.001 **
Right Atrium area, cm2, mean ± SD	15 ± 3.9	16 ± 6.2	15 ± 3.6	0.040	15 ± 4	15 ± 3	16 ± 6	16 ± 7	0.159
sPAP in Echo, mmHg, mean ± SD	27 ± 8	32 ± 13	26 ± 7	**<0.001**	26 ± 7	28 ± 7	28 ± 11	34 ± 15	**<0.001**
DETECT score, mean ± SD									
Step 1	312- ± 14	325 ± 21	310 ± 12	**< 0.001**	310 ± 12	311 ± 12	319 ± 20	330 ± 21	**<0.001**
Step 2	33 ± 8	41 ± 14	32 ± 7	**< 0.001**	32 ± 7	33 ± 8	37 ± 13	43 ± 14	**<0.001**
DETECT positive, *n* (%)	188 (34)	36 (61)	152 (31)	**< 0.001**	89 (30)	63 (32)	9 (39)	27 (75)	**< 0.001**
PH Groups (*n* = 80)									**<0.001**
RHC negative	42 (52)	4 (29)	38 (68)	**0.009**	19 (61)	19 (54)	1 (20)	3 (33)	
Precapillary PH	28 (35)	9 (64)	19 (34)		5 (16)	14 (40)	3 (60)	6 (67)	
Postcapillary PH	10 (13)	1 (7)	9 (16)		7 (23)	2 (6)	1 (20)	0	

Significant findings are highlighted in bold. Significance is set to 0.05.

* Student’s t test;

** Anova.

ACA: anti-centromere antibodies; ARA: anti-RNA polymerase III antibodies; ATA: anti-topoisomerase antibodies; CPFE: combined pulmonary fibrosis and emphysema; DLCO: diffusion capacity of the lung for carbon monoxide; FVC: functional vital capacity; LVEF: left ventricular ejection fraction; mRSS: modified Rodnan’s skin score; NYHA: New York Heart Association Function Classification; PH: pulmonary hypertension; RHC: right-heart catheterization; sPAP: estimated systolic pulmonary pressure in echocardiography.

### Emphysema and CPFE patients in our cohort

Among the selected cases, we identified 59 (10.7%) patients with emphysema on HRCT. As expected, SSc patients with emphysema were significantly older and more often ever smokers than non-emphysema cases. We observed significantly lower DLCO and higher FVC/DLCO ratio values in emphysema patients (*P < *0.001). Interestingly, emphysema patients presented significantly more often with concomitant ILD. They also presented with more prevalent vascular involvement characterized by more frequent digital ulcers and telangiectasia, as well as a higher estimated systolic pulmonary artery pressure (sPAP) on echocardiography and uric acid serum levels.

**Figure 1.**
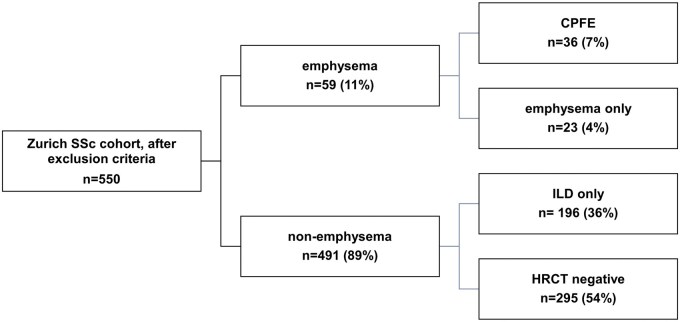
Study population segregated by ILD and/or emphysema. CPFE: combined pulmonary fibrosis and emphysema; ILD: interstitial lung disease

Among the emphysema patients, 36 (61%) were classified as CPFE, leaving 23 as ‘emphysema only’. Within the non-emphysema group, 196 individuals were considered ‘ILD only’ and 295 patients were HRCT negative (Fig. 1). CPFE patients were generally older, with higher prevalence of digital ulcers and telangiectasis and higher uric acid levels. Additionally, they presented with low FVC and disproportionately low DLCO, which led to a higher FVC/DLCO ratio. CPFE patients also showed high sPAP values on echocardiography. Patients with isolated emphysema also presented with impaired PFTs, elevated sPAP on echocardiogram and New York Heart Association (NYHA) functional class, resembling the patients with isolated ILD cases but not the negative HRCT group. Additional information is presented in Table 1.

### Association of emphysema and CPFE with the DETECT score

In line with our hypothesis, we observed that both DETECT score step 1 and step 2 were significantly higher in emphysema patients (*P < *0.001, see Supplementary Tables S1–S3). In contrast to non-emphysema patients, the mean DETECT score step 2 in emphysema patients was 41 points, exceeding the cut-off for referral to RHC (>35 points) (Table 1). This difference was confirmed when dividing the population in the four above-mentioned groups, where both emphysema-only and CPFE patients showed an average DETECT step 2 score above the referral threshold, with even higher values in the CPFE group.

Using linear regression modelling adjusted for confounders, we showed that the four groups were significantly associated with both DETECT step 1 (beta coefficient 3.11, 95% CI from 1.40–4.83, *P < *0.001) and step 2 (beta coefficient 1.76, 95% CI from 1.00–2.52, *P < *0.001) scores. When replacing the predictor CPFE with emphysema and ILD as separate variables, only emphysema (beta coefficient: 12.95, 95% CI 8.52–17.38, *P < *0.001) but not ILD (beta coefficient −2.39, 95% CI from −5.28–0.51 *P = *0.106) was associated with an increasing DETECT step 1 score. Similar results were obtained for the DETECT step 2 score (Table 2). In fact, the presence of emphysema on HRCT was independently associated with an increase of DETECT score step 2 of 6.22 points (*P < *0.001). Furthermore, increasing age, digital ulcers, male sex, diastolic dysfunction and a reduction in LVEF increased the DETECT score significantly (Table 2).

**Table 2.** Multivariable linear regression model to predict DETECT step 2 (ILD and emphysema as separate predictors)

	Regression coefficient (95% CI)	*P*-value
Constant	29.38 (21.41–37.35)	**<0.001 **
ILD	−0.45 (−1.75–0.83)	0.493
Emphysema	6.22 (4.24–8.20)	**<0.001 **
Age	0.23 (0.18–0.0.28)	**<0.001 **
Disease duration	−0.03 (−0.09–0.04)	0.438
Digital ulcers	2.67 (1.27–4.06)	**<0.001 **
Male sex	1.74 (0.08–3.40)	**0.040 **
LVEF, %	−0.18 (−0.30–0.05)	**0.006 **
Diastolic dysfunction	1.93 (0.26–3.59)	**0.023 **

Significant findings are highlighted in bold.

ILD: interstitial lung disease; LVEF: left ventricular ejection fraction.

### Positive *vs* negative DETECT scores

The DETECT step 2 score was positive in 188 individuals (34%): these patients were significantly older (*P < *0.001), more often ever-smokers (*P = *0.008) and had more frequently digital ulcers (*P < *0.001). In line with the previous results, we observed a higher prevalence of both emphysema and CPFE patients among cases with positive DETECT step 2 score (each *P < *0.001) (Table 3).

**Table 3.** Comparing DETECT step2 positive vs negative patients

	DETECT step 2 positive (*n* = 188)	DETECT step 2 negative (*n* = 362)	*P*-value^*^
Age, years, mean ± SD	64 ± 13	52 ± 14	**<0.001**
Sex female, *n* (%)	153 (81)	304 (84)	0.472
Disease duration, years, mean ± SD	10 ± 10	9 ± 9	0.080
Smoking ever, *n* (%)	63 (35)	84 (24)	**0.008**
Diffuse cutaneous SSc, *n* (%)	35 (19)	69 (19)	0.909
mRSS, median (IQR)	2 (0–6)	0 (0–4)	**0.028**
Digital ulcers ever, *n* (%)	71 (38)	88 (24)	**<0.001**
Puffy fingers, *n* (%)	138 (76)	243 (70)	0.219
Telangiectasia, *n* (%)	157 (84)	127 (35)	**<0.001**
Arthritis, *n* (%)	37 (20)	61 (17)	0.480
Esophageal symptoms, *n* (%)	112 (60)	167 (46)	**0.003**
Renal crisis, *n* (%)	5 (3)	10	**0.019**
ACA, *n* (%)	98 (56)	147 (43)	**0.005**
ATA, *n* (%)	35 (19)	102 (29)	**0.016**
ARA, *n* (%)	21 (12)	47 (13)	0.586
Diastolic dysfunction, *n* (%)	53 (29)	46 (13)	**<0.001 **
Uric acid (mg/dl), mean ± SD	5.7 ± 1.7	4.4 ± 1.1	**<0.001**
FVC, % predicted, mean ± SD	90 ± 22	95 ± 17	**0.019**
DLCO, % predicted, mean ± SD	63 ± 22	80 ± 20	**<0.001**
FVC/DLCO	1.64 ± 0.70	1.23 ± 0.28	**<0.001**
ILD, *n* (%)	90 (50)	142 (39)	0.056
Emphysema, *n* (%)	36 (19)	23 (6)	**<0.001**
CPFE, *n* (%)	27 (14)	9 (3)	**<0.001**
NYHA, *n* (%)			**<0.001 **
I	72 (39)	247 (69)	
II	86 (46)	98(28)	
III	23 (12)	11 (3)	
IV	6 (3)	1 0	
NT-proBNP, median (IQR)	248 (110–560)	93 (53–184)	**<0.001**
LVEF in %, mean ± SD	60 ± 6	61 ± 4	**0.045**
Right axis deviation, *n* (%)	5 (3)	0	**<0.001 **
Right Atrium area in cm2, mean ± SD	17 ± 5	14 ± 3	**<0.001**
sPAP on Echo, mmHg, mean ± SD	30 ± 10	25 ± 6	**<0.001**

Significant findings are highlighted in bold. Significance is set to 0.05;

* Student’s *t* test.

ACA: anti-centromere antibodies; ARA: anti-RNA polymerase III antibodies; ATA: anti-topoisomerase antibodies; CPFE: combined pulmonary fibrosis and emphysema; DLCO: diffusion capacity of the lung for carbon monoxide; FVC: functional vital capacity; ILD: interstitial lung disease; LVEF: left ventricular ejection fraction; mRSS: modified Rodnan’s skin score; NYHA: New York Heart Association Function Classification; sPAP: estimated systolic pulmonary pressure in echocardiography.

When predicting a positive DETECT step 2 score in logistic regression model, we observed that CPFE was not associated with a higher risk *per se* (OR 1. 23, 95% CI 0.96–1.58; Supplementary Table S4). Conversely, when repeating the model after separating ILD and emphysema, we observed that increasing age (OR 1.07, 95% CI 1.05–1.09, *P < *0.001), digital ulcers (OR 2.06, 95% CI 1.29–3.29, *P = *0.024), emphysema (OR 2.44, 95% CI 1.28–4.67, *P = *0.007) and a reduced ejection fraction (OR 0.96, 95% CI 0.92–1.00, *P = *0.035) were significantly associated with DETECT step 2 score positivity. Interestingly, ILD did not result independently associated with the outcome (Supplementary Table S5).

### True positive *vs* false-positive DETECT scores

**Figure 2.**
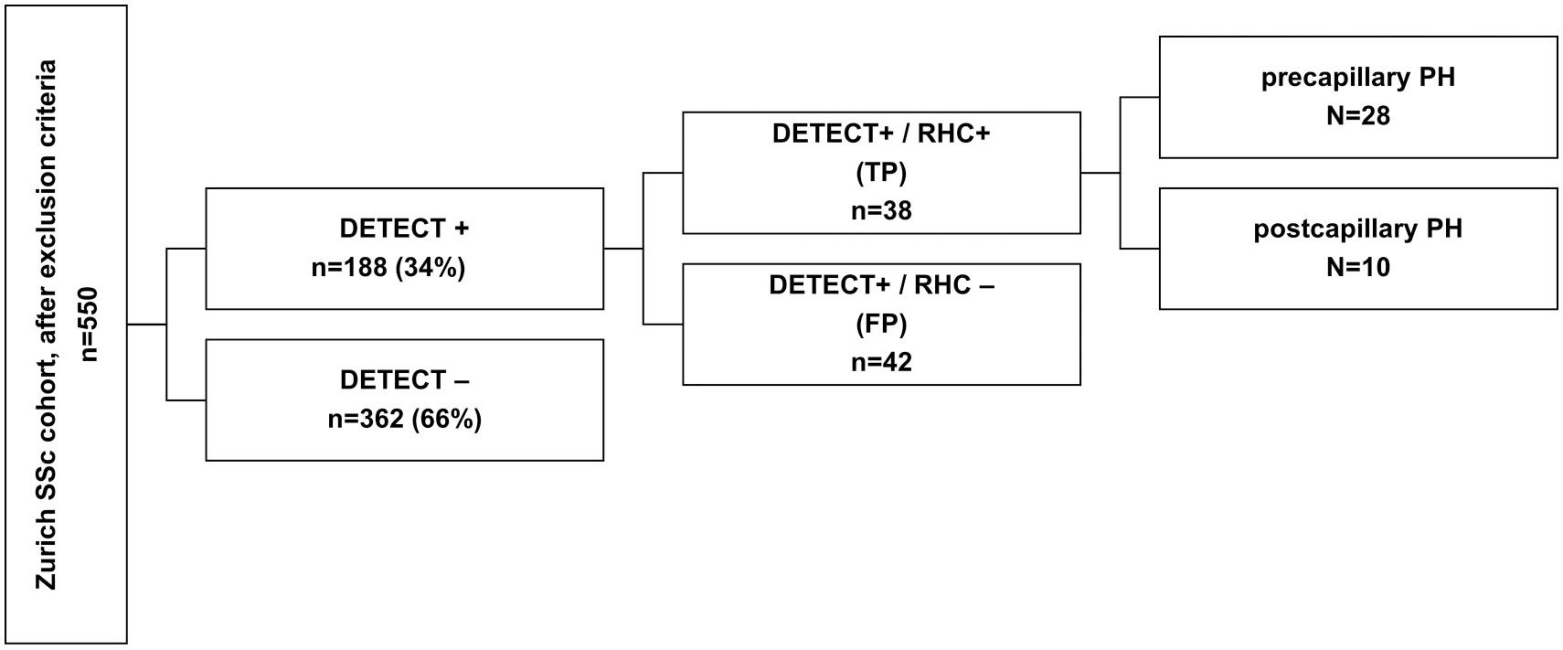
Study population segregated by DETECT positive/negative patients and RHC. DETECT+, DETECT score positive ( = above the threshold for RHC). DETECT−: DETECT score negative ( = below the threshold for RHC); PH: pulmonary hypertension; RHC: right-heart catheterization

RHC was performed in 80/188 (43%) patients with positive DETECT step 2 score. For patients who did not receive RHC, multidisciplinary discussion and additional non-invasive assessments (including cardiopulmonary exercise test (CPET), as well as consideration of previous negative RHC and patient preferences) were often determinant in taking the decision. In 38/80 (48%) cases of RHC performed, the diagnosis of PH was confirmed. Within this subgroup, 28 precapillary PH patients were identified (Fig. 2), of which 19 presented with PAH (Supplementary Table S6). Conversely, PH was excluded in 42 patients (DETECT step 2 false-positive) (Fig. 2). Comparing false-positive and true positive cases, we did not observe demographic, clinical or functional differences between the two groups aside from parameters reflecting PH: specifically sPAP, the DETECT step 2 score and its components (i.e. DLCO, FVC, NTproBNP) (Table 4). Still, in individuals with a DLCO of >60% false-positive DETECT scores were numerically more frequent, independently from disease duration (Supplementary Fig. S1). When predicting false-positive DETECT step 2 results through logistic regression, none of the variable tested demonstrated a statistically significant association (Supplementary Table S7). Specifically, the presence of emphysema (OR: 0.46, 95% CI 0.13–1.70, *P = *0.244), ILD (OR: 1.03, 95% CI 0.36–2.99) and CPFE (OR: 0.64, 95% CI 0.14–2.88) were not linked to a false-positive result.

**Table 4.** Comparison between true and false-positive DETECT score/right-heart catheterization results

	All DETECT step 2 positive, with RHC performed (*n* = 80)	DETECTstep 2 positive/RHC positive (*n* = 38)	DETECTstep 2 positive/RHC negative (*n* = 42)	*P*-value
Age, years, mean ± SD	64 ± 11	64 ± 12	65 ± 11	0.764
Sex female, *n* (%)	41 (72)	24 (73)	17 (71)	>0.999
Disease duration, years, mean ± SD	12 ± 12	11 ± 13	12 ± 12	0.846
Smoking ever, *n* (%)	22 (39)	12 (38)	10 (42)	0.788
Diffuse cutaneous SSc, *n* (%)	13 (23)	10 (31)	3 (13)	0.200
mRSS, median (IQR)	2 (0–6)	4 (0–9)	3 (2–6)	0.770
Digital ulcers ever, *n* (%)	25 (44)	14 (42)	11 (46)	>0.999
Puffy fingers, *n* (%)	45 (80)	24 (75)	21 (88)	0.319
Telangiectasia, *n* (%)	50 (88)	28 (85)	22 (92)	0.687
Arthritis, *n* (%)	5 (9)	2 (6)	3 (13)	0.640
Esophageal symptoms, *n* (%)	34 (59)	22 (67)	12 (50)	0.276
Renal crisis, *n* (%)	1 (2)	1 (3)	0	>0.999
ACA/*n* (%)	24 (44)	14 (44)	10 (44)	>0.999
ATA/*n* (%)	9 (16)	6 (18)	3 (13)	0.723
ARA/*n* (%)	5 (9)	2 (6)	3 (14)	0.379
Diastolic dysfunction, *n* (%)	18 (32)	8 (25)	10 (42)	0.250
Uric acid (mg/dl), mean ± SD	6.3 ± 2.3	6.6 ± 2.7	6.0 ± 1.8	0.360
FVC, % predicted, mean ± SD	85 ± 21	80 ± 23	92 ± 17	**0.034**
DLCO, % predicted, mean ± SD	53 ± 20	45 ± 18	63 ± 19	**<0.001**
FVC/DLCO	1.9 ± 0.8	2.1 ± 0.9	1.6 ± 0.5	**0.023**
ILD, *n* (%)	33 (58)	19 (58)	14 (58)	>0.999
Emphysema, *n* (%)	14 (25)	10 (30)	4 (17)	0.352
CPFE, *n* (%)	9 (16)	6 (18)	3 (13)	0.723
NT-proBNP, median (IQR)	247 (110–560)	446 (166–1774)	255 (114–495)	**0.049**
LVEF in %, mean ± SD	60 ± 6	59 ± 7	61 ± 5	0.263
Right axis deviation, *n* (%)	2 (<1)	1 (<1)	1 (<1)	>0.999
Right Atrium area, cm2, mean ± SD	18 ± 6	18 ± 6	18 ± 6	0.792
sPAP on Echo, mmHg, mean ± SD	33 ± 11	37 ± 13	28 ± 5	**0.001**

Significant findings are highlighted in bold.

ACA: anti-centromere antibodies; ARA: anti-RNA polymerase III antibodies; ATA: anti-topoisomerase antibodies; CPFE: combined pulmonary fibrosis and emphysema; DETECTs2: DETECT score step 2; DLCO: diffusion capacity of the lung for carbon monoxide; FVC: functional vital capacity; LVEF: left ventricular ejection fraction; mRSS: modified Rodnan’s skin score; NYHA: New York Heart Association Function Classification; PH: pulmonary hypertension; RHC: right-heart catheterization; sPAP: estimated systolic pulmonary pressure in echocardiography.

## Discussion

Our study demonstrated that emphysema and CPFE can complicate the picture of SSc and are linked to a significant increase in the DETECT score. Despite this, and the higher prevalence of DETECT score positivity, neither CPFE nor emphysema nor ILD were associated with false-positive DETECT step 2 scores. This translates to the applicability of the DETECT algorithm in SSc emphysema patients with and without ILD, not causing unnecessary RHC procedures.

SSc carries a high morbidity and mortality burden, to which cardiopulmonary involvement contributes extensively [1]. In addition to PH and ILD, airway diseases can complicate SSc cases, either presenting as isolated entities, like emphysema, or in concomitance with pulmonary fibrosis. CPFE patients, in particular, have been shown to carry a higher risk for unfavourable outcomes, in terms of both PFTs impairment and mortality [4, 5, 21].

Our cohort study analyses in-depth whether the decline of PFTs values associated to CPFE/emphysema affects the inclusion of these parameters in the DETECT algorithm screening for PH. Screening for PH has been of interest to the scientific community for the past two decades [22]. Among the available algorithms, the DETECT score has emerged for its multi-domain nature, high sensitivity and broad validation in real-life scenarios [23, 24]. Given that the annual application of the DETECT in SSc patients is part of the most recent international recommendations for the management of PH [16], it is pivotal to confirm the applicability of such procedures also to SSc patients affected by other organ involvements. Originally, the DETECT score was validated for patients presenting with disease duration >3 years and a DLCO <60% [11] and was later supported in an early SSc cohort with DLCO >60%, including also patients with short disease duration [17]. The good performance of the DETECT score has been additionally confirmed to identify patients affected by pulmonary hypertension according to the new haemodynamic definition [23].

However, its application was not investigated among patients with emphysema and CPFE, both determining a decline of DLCO and increase of the FVC/DLCO ratio, which influences the DETECT final score [24], as well as in patients with various extents of left heart disease.

Our study population showed a prevalence of emphysema of 10.7%, which increased to 15.5% when specifically mentioning CPFE among SSc ILD patients, being in line with previous data within the unselected SSc cohort [4–6]. Similarly, while the prevalence of some extent of diastolic dysfunction was in line with previous reports, the prevalence of left-heart systolic dysfunction was neglectable.

We could observe that both DETECT step 1 and step 2 scores were significantly higher in emphysema patients, even when separated into isolated emphysema and CPFE cases. Although PFTs might play a major role in these results, NT-proBNP and uric acid serum levels were also higher in SSc patients with emphysema and CPFE, telangiectasia occurred more often in both subgroups, whereas ACA was more frequent in emphysema only patients. These findings demonstrate that various compounds included in DETECT step 1 and 2 scores might lead to the increase in DETECT scores in emphysema SSc patients, which is not only dependent on PFTs changes related to airways disease.

When applying the cut-off for recommending referral to RHC after the DETECT step 2 (>35 points), we observed a significantly higher prevalence of both CPFE and emphysema in the DETECT step 2 positive group. Although ILD also showed a higher prevalence in the DETECT step 2 positive group, there was only a trend towards statistical significance as an independent predictor. These results were reflected by our regression models, in which emphysema was ‘superior’ to ILD as an independent predictor of positive DETECT step 2 scores. A possible explanation could be related to the differences in FVC/DLCO ratio between isolated ILD patients *vs* isolated emphysema/CPFE groups. In fact, FVC/DLCO ratio in ILD patients was comparable to that of SSc patients without pulmonary manifestations, whereas CPFE or emphysema patients presented with an elevated FVC/DLCO ratio (1.37 in ILD patients *vs* 1.61 in emphysema/2.15 in CPFE patients). Consistently, uric acid levels were lower in the ILD group compared with the CPFE or emphysema groups, and ILD patients were less frequent ACA positive, further contributing to a lower DETECT score.

The link between emphysema and PH is well known and finds different supportive explanations also in SSc patients. In fact, the spectrum of PH in SSc is very heterogeneous including pulmonary arterial hypertension (vasculopathy of the pulmonary arteries, PH group I), PH due to left heart disease (PH group II) or secondary to lung disease (PH group III) [25]. Emphysema is a long-time recognized risk factor for the development of group III PH and might further increase the risk for PH in addition to ILD [26]. In the present study, we demonstrated that in CPFE and emphysema patients who underwent RHC following a positive DETECT step 2 score, the diagnosis of PH was confirmed in 67% and 80% of cases respectively, which was significantly more frequent than in the overall study population (48%). These numbers support the hypothesis that PH is more frequent in emphysema and CPFE patients, leading to a higher chance of true positive DETECT scores in these subgroups. In line with the previous argumentation, neither CPFE (*P = *0.563) nor emphysema (*P = *0.244) were associated with false-positive DETECT score. However, the latter should be regarded with caution, as only half of the DETECT positive cases in our cohort underwent RHC as formally indicated by recommendations.

Interestingly, patients with isolated ILD presented on average with a normal DETECT step 2 score, not exceeding the threshold of 35 points, in contrast to emphysema and CPFE patients. Despite the well-known coexistence of PH and ILD (ILD among SSc PH amounting to ∼21%) [27], our results did not show ILD as an independent risk factor for increasing the DETECT score, nor as a predictor for false-positive results. A possible explanation might be found in the PFTs measurements. In fact, the FVC/DLCO ratio is typically not elevated in ILD patients, given the concomitant reduction of both FVC and DLCO [28]. Further, it could be argued that the presence of SSc-ILD is more frequently linked to ATA positivity and the absence of ACA, an immunologic constellation which might carry a certain protective effect concerning the development of PH [29]. In fact, both the average DETECT score, the number of DETECT step 2 score positive cases and the final percentage of true positive cases was comparable to the group with negative HRCT. Therefore, our results support the applicability of the DETECT score also in SSc patients with ILD, in whom the performance of the screening algorithm was comparable to that of the ILD negative population.

The strength of this study is the real-word data of our well characterized and prospectively followed SSc cohort, with detailed HRCT, PFTs, demographic and clinical data. Furthermore, although relatively uncommon, the number of CPFE/emphysema patients in our cohort is notable, enhancing the power of our statistical analysis.

Still, our study is not without limitations. First, the number of RHC performed in relation to DETECT positive patients is lower than expected. This might be related to the retrospective nature of the study, through which we calculated the DETECT score also to visits performed before the algorithm was published and implemented in local clinical practice. In addition, our centre follows the up-to-date practice of multidisciplinary case discussion, which has been shown to improve the selection of positive RHC cases and the specificity of the screening procedures [30]. This also includes the application of additional, non-invasive evaluations, such as cardiopulmonary exercise test, which has been shown to improve the accuracy of the DETECT algorithm [31]. Overall, we observed that very few additional cases of PH were detected during the follow-up of the same cohort, both among initially DETECT negative (three PH diagnosis of four RHC performed) and initially DETECT positive who did not undergo RHC (two PH diagnosis of three RHC performed). This stresses the need to continue screening for PH over time, even after a first negative assessment.

Second, the diagnosis of emphysema and ILD were performed by experienced thoracic radiologists at the time of the clinical consultation and based on HRCT images following current definitions and recommendations [18]. Still, we did not perform a centralized reading of the scans and did not include quantification of the extents of ILD and emphysema in our analysis. This might have indeed missed the opportunity to identify specific connection between these factors and DETECT positivity.

Third, DETECT algorithm has been developed for screening pulmonary arterial hypertension (PH group I), intentionally enriching for group I risk factors and excluding cases with severe suspicion for group 2 or group 3 PH. However, clinical practice also includes these scenarios. In particular, it is clinically challenging to fully identify the aetiology of respiratory symptoms, specifically in a multisystem disease like SSc. In fact, dyspnea may reflect both the severity of underlying lung disease, as expression of emphysema or ILD, as well as pulmonary vascular involvement in terms of PH, or heart disease. In this study, reflecting our clinical practice, we applied DETECT score for the screening of any PH form, including also its use in symptomatic patients, further supporting the referral to RHC. We acknowledge that this extends beyond the scope of current guidelines-directed screening recommendations, but we believe that this approach may reflect real-world clinical scenarios where PH due to left heart or lung disease is also frequently encountered in SSc patients and contributes significantly to morbidity. We believe that early detection of PH in these populations remains clinically meaningful. In group 2 PH, early detection may prompt optimization of heart failure management. For group 3 PH, particularly in CPFE or severe emphysema, identifying PH could support earlier referral for lung transplantation evaluation, given the lack of effective medical therapies.

To conclude, our analysis provides evidence that the DETECT score is significantly elevated in emphysema and CPFE patients. Emphysema, CPFE and ILD do not determine unneeded referral to RHC procedures. On the contrary, our data support the usefulness and the applicability of the DETECT score also in patients with ILD, emphysema or CPFE, where the main mechanisms leading to PH might be different from the pure arterial disease.

## Supplementary material

Supplementary material is available at *Rheumatology* online.

## Supplementary Material

keaf410_Supplementary_Data

## Data Availability

Anonymized data can be made available upon reasonable request to the corresponding author.

## References

[1] Elhai M , Meune C, Boubaya M et al; EUSTAR Group. Mapping and predicting mortality from systemic sclerosis. Ann Rheum Dis 2017;76:1897–905. 28835464 10.1136/annrheumdis-2017-211448

[2] Lin H , Jiang S. Combined pulmonary fibrosis and emphysema (CPFE): an entity different from emphysema or pulmonary fibrosis alone. J Thorac Dis 2015;7:767–79. 25973246 10.3978/j.issn.2072-1439.2015.04.17 PMC4419325

[3] Cottin V , Nunes H, Brillet P-Y et al Combined pulmonary fibrosis and emphysema: a distinct underrecognised entity. Eur Respir J 2005;26:586–93. 16204587 10.1183/09031936.05.00021005

[4] Ariani A , Silva M, Bravi E et al Overall mortality in combined pulmonary fibrosis and emphysema related to systemic sclerosis. RMD Open 2019;5:e000820. 30886735 10.1136/rmdopen-2018-000820 PMC6397433

[5] Champtiaux N , Cottin V, Chassagnon G et al Combined pulmonary fibrosis and emphysema in systemic sclerosis: a syndrome associated with heavy morbidity and mortality. Semin Arthritis Rheum 2019;49:98–104. 30409416 10.1016/j.semarthrit.2018.10.011

[6] Antoniou KM , Margaritopoulos GA, Goh NS et al Combined pulmonary fibrosis and emphysema in scleroderma-related lung disease has a major confounding effect on lung physiology and screening for pulmonary hypertension. Arthritis Rheumatol 2016;68:1004–12. 26636545 10.1002/art.39528

[7] Smith H , Thompson AAR, Akil M et al The spectrum of systemic sclerosis-associated pulmonary hypertension: insights from the ASPIRE registry. J Heart Lung Transplant 2024;43:1629–39. 39260921 10.1016/j.healun.2024.06.007

[8] Hage R , Gautschi F, Steinack C, Schuurmans MM. Combined Pulmonary Fibrosis and Emphysema (CPFE) clinical features and management. Int J Chron Obstruct Pulmon Dis 2021;16:167–77. 33536752 10.2147/COPD.S286360 PMC7850450

[9] Steen V , Medsger TA. Predictors of isolated pulmonary hypertension in patients with systemic sclerosis and limited cutaneous involvement. Arthritis Rheum 2003;48:516–22. 12571862 10.1002/art.10775

[10] Colalillo A , Hachulla E, Pellicano C et al Diffusing capacity of the lungs for carbon monoxide and echocardiographic parameters in identifying mild pulmonary hypertension in the EUSTAR cohort of patients with systemic sclerosis. Chest 2024;166:837–44. 38849072 10.1016/j.chest.2024.05.010

[11] Coghlan JG , Denton CP, Grünig E et al; DETECT Study Group. Evidence-based detection of pulmonary arterial hypertension in systemic sclerosis: the DETECT study. Ann Rheum Dis 2014;73:1340–9. 23687283 10.1136/annrheumdis-2013-203301 PMC4078756

[12] Giucă A , Mihai C, Jurcuț C et al Screening for pulmonary hypertension in systemic sclerosis-a primer for cardio-rheumatology clinics. Diagnostics (Basel) 2021;11:1013. 34206055 10.3390/diagnostics11061013 PMC8229459

[13] Thakkar V , Stevens WM, Prior D et al N-terminal pro-brain natriuretic peptide in a novel screening algorithm for pulmonary arterial hypertension in systemic sclerosis: a case-control study. Arthritis Res Ther 2012;14:R143. 22691291 10.1186/ar3876 PMC3446526

[14] Lepri G , Bruni C, Tofani L et al The performance of pulmonary function tests in predicting systemic sclerosis-interstitial lung disease in the european scleroderma trial and research database. Diagnostics (Basel) 2024;14:295. 38337811 10.3390/diagnostics14030295 PMC10855256

[15] Bruni C, , Tofani L, , Fretheim H, , Distler O. A screening tool to detect interstitial lung disease in systemic sclerosis: the ILD-RISC Score. Rheumatology (Oxford) 2025;in press. 10.1093/rheumatology/keaf445 PMC12671881 40811030

[16] Humbert M , Kovacs G, Hoeper MM et al; ESC/ERS Scientific Document Group. 2022 ESC/ERS Guidelines for the diagnosis and treatment of pulmonary hypertension. Eur Heart J. 11. Oktober 2022;43:3618–731. 10.1093/eurheartj/ehac237 36017548

[17] Young A , Moles VM, Jaafar S et al Performance of the DETECT algorithm for pulmonary hypertension screening in a systemic sclerosis cohort. Arthritis Rheumatol 2021;73:1731–7. 33760392 10.1002/art.41732 PMC8403104

[18] Bankier AA , MacMahon H, Colby T et al Fleischner Society: glossary of Terms for Thoracic Imaging. Radiology 2024;310:e232558. 38411514 10.1148/radiol.232558 PMC10902601

[19] Cacciapaglia F , De Angelis R, Ferri C et al; Systemic Sclerosis Progression Investigation Group of the Italian Society for Rheumatology (SPRING-SIR). Pulmonary Arterial Hypertension Incidence in Patients With Systemic Sclerosis Treated With Bosentan for Digital Ulcers: evidence From the SPRING-SIR Registry. J Rheumatol 2025;52:375–82. 39814435 10.3899/jrheum.2024-0750

[20] Nagueh SF , Smiseth OA, Appleton CP et al Recommendations for the evaluation of left ventricular diastolic function by echocardiography: an update from the American Society of Echocardiography and the European Association of Cardiovascular Imaging. J Am Soc Echocardiogr 2016;29:277–314. 27037982 10.1016/j.echo.2016.01.011

[21] Koo BS , Park KY, Lee HJ et al Effect of combined pulmonary fibrosis and emphysema on patients with connective tissue diseases and systemic sclerosis: a systematic review and meta-analysis. Arthritis Res Ther 2021;23:100. 33823923 10.1186/s13075-021-02494-y PMC8022385

[22] Bruni C , De Luca G, Lazzaroni M-G et al Screening for pulmonary arterial hypertension in systemic sclerosis: a systematic literature review. Eur J Intern Med 2020;78:17–25. 32540411 10.1016/j.ejim.2020.05.042

[23] Distler O , Bonderman D, Coghlan JG et al Performance of DETECT pulmonary arterial hypertension algorithm according to the hemodynamic definition of pulmonary arterial hypertension in the 2022 European Society of Cardiology and the European Respiratory Society Guidelines. Arthritis Rheumatol 2024;76:777–82. 38146100 10.1002/art.42791

[24] Guillén-Del Castillo A , Callejas-Moraga EL, García G et al High sensitivity and negative predictive value of the DETECT algorithm for an early diagnosis of pulmonary arterial hypertension in systemic sclerosis: application in a single center. Arthritis Res Ther 2017;19:135. 28615037 10.1186/s13075-017-1327-8 PMC5471690

[25] Bruni C , Guignabert C, Manetti M, Cerinic MM, Humbert M. The multifaceted problem of pulmonary arterial hypertension in systemic sclerosis. Lancet Rheumatol 2021;3:e149–59–e159. 38279370 10.1016/S2665-9913(20)30356-8

[26] Jankowich MD , Rounds SIS. Combined pulmonary fibrosis and emphysema syndrome: a review. Chest 2012;141:222–31. 22215830 10.1378/chest.11-1062 PMC3251269

[27] Hinchcliff M , Fischer A, Schiopu E, Steen VD; PHAROS Investigators. Pulmonary Hypertension Assessment and Recognition of Outcomes in Scleroderma (PHAROS): baseline characteristics and description of study population. J Rheumatol 2011;38:2172–9. 21844142 10.3899/jrheum.101243 PMC3230328

[28] Caron M , Hoa S, Hudson M, Schwartzman K, Steele R. Pulmonary function tests as outcomes for systemic sclerosis interstitial lung disease. Eur Respir Rev 2018;27:170102. 29769294 10.1183/16000617.0102-2017 PMC9488607

[29] Khanna D , Tashkin DP, Denton CP et al Etiology, risk factors, and biomarkers in systemic sclerosis with interstitial lung disease. Am J Respir Crit Care Med 2020;201:650–60. 31841044 10.1164/rccm.201903-0563CI PMC7068837

[30] Coirier V , Chabanne C, Jouneau S et al Impact of three different algorithms for the screening of SSc-PAH and comparison with the decisions of a multidisciplinary team. Diagnostics (Basel) 2021;11:1738. 34679436 10.3390/diagnostics11101738 PMC8534432

[31] Santaniello A , Casella R, Vicenzi M et al Cardiopulmonary exercise testing in a combined screening approach to individuate pulmonary arterial hypertension in systemic sclerosis. Rheumatology (Oxford) 2020;59:1581–6. 31637433 10.1093/rheumatology/kez473 PMC7310101

