# Supplementary material for: Emphysema elevates the DETECT scores: impact on pulmonary hypertension screening and diagnosis in systemic sclerosis
Source: Rheumatology (Oxford). 2025 Aug 1;64(12):6132–41. doi: 10.1093/rheumatology/keaf410 (PMC12671872; doi:10.1093/rheumatology/keaf410)
Supplement: keaf410_Supplementary_Data [file keaf410_supplementary_data.docx]

**Supplementary material**

**Supplementary Table S1: Multivariable linear regression model for DETECT score step 1 (CPFE as a single predictor).**

|  | **regression coefficient (95% CI)** | **p-value** |
| --- | --- | --- |
| Constant | 293 (275 - 311) | **<0.001** |
| CPFE (4 groups) | 3.11 (1.40 - 4.83) | **<0.001** |
| Age | 0.53 (0.42 - 0.63) | **<0.001** |
| Disease duration | -0.07 (-0.22 - 0.85) | 0.396 |
| Digital ulcers | 4.84 (1.71 - 7.97) | **0.003** |
| Sex | 2.26 (-1.53 - 6.05 | 0.241 |
| LVEF % | -0.29 (-0.58 - -0.01) | **0.044** |
| Diastolic dysfunction | 4.71 (0.93 - 8.49) | **0.015** |

Significant findings are highlighted in bold**.** CI: confidence interval; CPFE, combined pulmonary fibrosis and emphysema; LVEF, left ventricular ejection fraction.

**Supplementary Table S2: Multivariable linear regression model for DETECT score step 1 (ILD and emphysema as separate predictors).**

|  | **regression coefficient (95% CI)** | **p-value** |
| --- | --- | --- |
| Constant | 292 (274 - 310) | **<0.001** |
| ILD | -2.39 (-5.28 - 0.51) | 0.106 |
| Emphysema | 12.95 (8.52 - 17.38) | **<0.001** |
| Age | 0.53 (0.43 - 0.64) | **<0.001** |
| Disease duration | -0.06 (-0.20 - 0.09) | 0.453 |
| Digital ulcers | 6.14 (3.02 - 9.26) | **<0.001** |
| Sex | 2.64 (-1.08 - 6.36) | 0.165 |
| LVEF % | -0.27 (-0.55 - 0.01) | 0.061 |
| Diastolic dysfunction | 4.97 (1.26 - 8.69) | **0.009** |

Significant findings are highlighted in bold. CI: confidence interval; ILD, interstitial lung disease; LVEF, left ventricular ejection fraction.

**Supplementary Table S3: Multivariable linear regression models for DETECT score step 2 (CPFE as a single predictor).**

|  | **regression coefficient (95% CI)** | **p-value** |
| --- | --- | --- |
| Constant | 29.731 (21.64 -37.82) | **<.0001** |
| CPFE | 1.76 (1.00-2.52) | **<0.001** |
| Age | 0.23 (0.18 -0.28) | **<0.001** |
| Disease duration | -0.03 (-0.10 - 0.04) | 0.387 |
| Digital ulcers | 2.14 (0.75 -3.54) | **0.003** |
| Male Sex | 1.59 (-0.10 -3.28) | 0.065 |
| LVEF, % | -0.19 (-0.32- -0.06) | **0.004** |
| Diastolic dysfunction | 1.82 (0.13 -3.50) | **0.034** |

Significant findings are highlighted in bold letters. CI: confidence interval; CPFE, combined pulmonary fibrosis and emphysema; LVEF, left ventricular ejection fraction.

**Supplementary Table S4: Risk factors for DETECT step 2 positivity** (**CPFE as a single predictor).**

| **Variables** | **Odds ratio** **(95% CI)** | **p-value** |
| --- | --- | --- |
| Age | 1.07 (1.05 - 1.09) | **<0.001** |
| Sex | 1.03 (0.60 - 1.79) | 0.907 |
| Disease duration | 0.99 (0.97 - 1.01) | 0.373 |
| Digital ulcers | 1.84 (1.17 – 2.89) | **0.008** |
| Smoking | 1.43 (0.92 - 2.24) | 0.116 |
| CPFE | 1.23 (0.96 – 1.58) | 0.101 |
| LVEF % | 0.96 (0.92 – 1.00) | **0.031** |
| Diastolic dysfunction | 1.38 (0.83 - 2.29) | 0.216 |

Results of a multivariable logistic regression model. Significant findings are highlighted in bold. CI: confidence interval; ILD, Interstitial lung disease; LVEF, left ventricular ejection fraction. OR: odds ratio.

**Supplementary Table S5: Risk factors for DETECT step 2 positivity (ILD and emphysema as separate predictors).**

| **Variables** | **Odds ratio** **(95% CI)** | **p-value** |
| --- | --- | --- |
| Age | 1.07 (1.05 - 1.09) | **<0.001** |
| Sex | 1.07 (0.62 - 1.87) | 0.807 |
| Disease duration | 0.99 (0.97 - 1.01) | 0.368 |
| Digital ulcers | 2.06 (1.29 - 3.29) | **0.024** |
| Smoking | 1.31 (0.83 - 2.07) | 0.246 |
| Emphysema | 2.44 (1.28 - 4.67) | **0.007** |
| ILD | 0.82 (0.53 - 1.26) | 0.363 |
| LVEF % | 0.96 (0.92 – 1.00) | **0.035** |
| Diastolic dysfunction | 1.39 (0.83 - 2.31) | 0.213 |

Results of a multivariable logistic regression model. Significant findings are highlighted in bold. CI: confidence interval; ILD, Interstitial lung disease; LVEF, left ventricular ejection fraction. OR: odds ratio

**Supplementary Table S6: Frequencies of PH groups according the 2014(1) and 2022(2) hemodynamic definition of pulmonary hypertension.**

| **Frequencies** | | **2022 ESC/ERS definition** | | | | |
| --- | --- | --- | --- | --- | --- | --- |
|  |  | **Non-PH** ^e^ | **WHO Group 1 PH (PAH)** ^f^ | **WHO Group 2 PH** ^g^ | **WHO Group 3 PH**^h^ | **Total** |
| **2014 definition** | **Non-PH** ^a^ | 42 | 2 | 1 | 1 | 46 |
|  | **WHO Group 1 PH (PAH)**^b^ | 0 | 17 | 0 | 0 | 17 |
|  | **WHO Group 2 PH**^c^ | 0 | 0 | 9 | 0 | 9 |
|  | **WHO Group 3 PH**^d^ | 0 | 0 | 0 | 8 | 8 |
|  | **Total** | 42 | 19 | 10 | 9 | 80 |

Subanalysis of all Patient in whom RHC was performed classified using the 2014 (1) and 2022 ESC/ERS definitions (2) (N = 80). ESC/ERS, European Society of Cardiology/European Respiratory Society; FVC, forced vital capacity; HRCT, high-resolution computed tomography; LHD, left heart disease; mPAP, mean pulmonary arterial pressure; PAH, pulmonary arterial hypertension; PCWP, pulmonary capillary wedge pressure; PH, pulmonary hypertension; PVR, pulmonary vascular resistance; WHO, World Health Organization; WU, Wood units.

^a^mPAP <25 mmHg.  ^b^mPAP ≥25 mmHg, PCWP ≤15 mm Hg plus HRCT showing no parenchymal lung disease. ^c^mPAP ≥25 mmHg and PCWP >15 mm Hg. ^d^mPAP ≥25 mmHg, PCWP ≤15 mm Hg plus HRCT showing parenchymal lung disease with ILD extent >20% or FVC<70%. ^e^ mPAP ≤20 mmHg. ^f^ mPAP >20 mmHg. PCWP ≤15 mm Hg, PVR >2 WU, plus HRCT showing no parenchymal lung disease.  ^g^mPAP >20 mmHg, PCWP >15 mm Hg ^h^mPAP >20 mmHg and PCWP≤15 mm Hg plus HRCT showing parenchymal lung disease with ILD extent >20% or FVC<70%.

References

1 Coghlan JG, Denton CP, Grünig E, Bonderman D, Distler O, Khanna D, u. a. Evidence-based detection of pulmonary arterial hypertension in systemic sclerosis: the DETECT study. Ann Rheum Dis. 1. Juli 2014;73(7):1340.

2 Distler O, Bonderman D, Coghlan JG, Denton CP, Grünig E, Khanna D, u. a. Performance of DETECT Pulmonary Arterial Hypertension Algorithm According to the Hemodynamic Definition of Pulmonary Arterial Hypertension in the 2022 European Society of Cardiology and the European Respiratory Society Guidelines. Arthritis Rheumatol. Mai 2024;76(5):777–82.

**Supplementary Table S7: Univariable logistic regression prediction model for false positive DETECT score (DETECT +/RHC-).**

|  | **Odds ratio (95% CI)**  **(95% CI)** | **p-value** |
| --- | --- | --- |
| Age | 1.01 (0.96 - 1.06) | 0.760 |
| LVEF | 1.05 (0.96 - 1.15) | 0.263 |
| Diastolic dysfunction | 2.14 (0.69 - 6.70) | 0.190 |
| Smoke | 1.19 (0.40 - 3.51) | 0.752 |
| Digital ulcers | 1.15 (0.40 - 3.31) | 0.529 |
| Emphysema | 0.46 (0.13 - 1.70) | 0.244 |
| CPFE | 0.64 (0.14 - 2.88) | 0.563 |
| ILD | 1.03 (0.36-2.99) | 0.954 |

Significant findings are highlighted in bold letters**.** CI: confidence interval; CPFE, combined pulmonary fibrosis and emphysema; ILD, interstitial lung disease; LVEF, left ventricular ejection fraction. RHC, right heart catheterization.

**Supplementary Figure S1: Distribution of patients, DETECT positive and pulmonary hypertension cases according to disease duration and DLCO.**


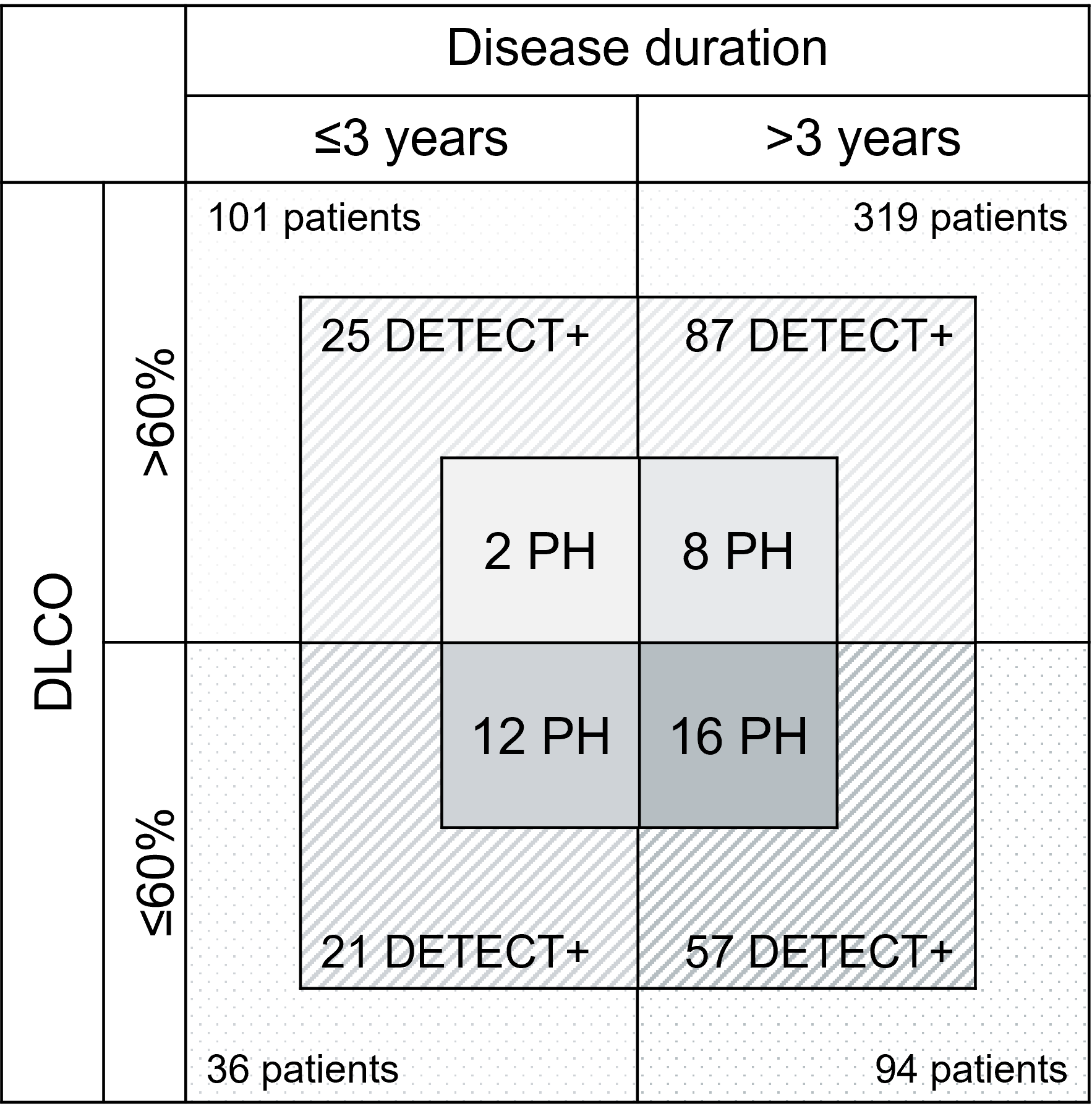


Legend: The bottom right section (disease duration over 3 years and DLCO ≤ 60%) is in line with the original DETECT study inclusion criteria. Dotted areas represent patients; striped areas DETECT positive cases, solid areas PH cases. DLCO: diffusion capacity of the lung for carbon oxide; PH: pulmonary hypertension.
